# Semantic and Phonemic Verbal Fluency in Parkinson’s Disease: Influence of Clinical and Demographic Variables

**DOI:** 10.3233/BEN-2011-0354

**Published:** 2012-01-20

**Authors:** Ignacio Obeso, Enrique Casabona, Maria Luisa Bringas, Lázaro Álvarez, Marjan Jahanshahi

**Affiliations:** ^1^Cognitive Motor Neuroscience GroupSobell Department of Motor Neuroscience & Movement DisordersUCL Institute of NeurologyLondonUK; ^2^Clínica de Trastornos del MovimientoCentro Internacional de Restauración NeurológicaCIRENCubanacánHabanaCuba

**Keywords:** Verbal fluency, phonemic, semantic, Parkinson's disease, executive function, cognition

## Abstract

Changes of cognitive function in PD have been extensively documented and defined as a ‘frontal’ type executive dysfunction. One of the main components of this executive dysfunction is the impairment of verbal fluency. The aim of the present study was to assess semantic and phonemic fluency in a large sample of PD patients and to investigate the effect of clinical and sociodemographic variables on verbal fluency in this patient group.

Three hundred patients with idiopathic Parkinson's disease who were consecutive referrals to our clinic and 50 age and education matched healthy controls completed the phonemic and semantic verbal fluency tasks. Both phonemic and semantic verbal fluency were significantly impaired in PD patients relative to matched controls. Stage of illness, presence of depression, education and age influenced verbal fluency measures. Regression analyses established that global measures of cognitive ability (MMSE) and executive function (FAB) and side of onset of motor symptoms predicted 36–37% of variance of phonemic or semantic verbal fluency measures. Thus, future studies aimed at assessing cognitive functioning in PD patients treated by deep brain stimulation (DBS) should adequately take into account several factors (stage of illness, depression, executive functioning) which may potentially influence performance on verbal fluency tasks.

## 1. Introduction

While historically Parkinson's disease has been considered as a motor disorder, the clinical importance of the non-motor symptoms such as cognitive deficits, depression, hallucinations and apathy are increasingly recognized, particularly as most of these non-motor symptoms have been shown to influence quality of life [1]. Disturbance of cognitive function in PD has now been extensively documented and defined as a 'frontal' type executive dysfunction [2]. One of the main components of this executive dysfunction is the impairment of verbal fluency, noted to be present from the initial stages of the illness [3]. Impairment of verbal fluency in PD is of key interest for two main reasons. First, it has been identified as a predictor of subsequent dementia in PD [4]. Second, increased deficits in verbal fluency is the only consistent and persistent cognitive impairment reported following deep brain stimulation of the subthalamic nucleus or the internal segment of the globus pallidus in PD (for review see [5]). Yet, relatively little information is available about how key clinical features of PD influence performance on verbal fluency tests in PD. The aim of the present cross-sectional study was to investigate the impact of key demographic (age, gender, education) and clinical variables such as age of onset, duration of illness, stage of illness, presence/absence of clinical depression on verbal fluency in a large group of patients with idiopathic PD.

ISSN 0953-4180/12/$27.50 © 2012 – IOS Press and the authors. All rights reserved

## 2. Methods

### 2.1. Sample

The patients were consecutive referrals to the Movement Disorders and Neurodegenerative Illness Clinic of the Centro Internacional de Restauracion Neurologica (CIREN) in Havana, Cuba, between January of 2001 and June of 2003. We assessed 300 (217 male; 292 right-handed) spanish-speaking patients with idiopathic PD diagnosed according to the UK Parkinson's Disease Brain Bank criteria [6]. The mean age was 57.95 years (*SD* = 10.7). An age cutoff of 58 years was used to compare younger and older patients. On the Hoehn and Yahr scale [7], the mean stage of illness was 2.6 (*SD* = 1.0), with a range of 1 to 4. The average duration of illness was 7.80 years (*SD* = 4.9). We used 10 years as a criterion for separating the sample into short vs. long duration of illness. The mean age at onset was 50.15 years (*SD* = 11.6). Using the age of onset cutoff of 50 [8], early vs. late onset subgroups were defined. The average years of education for the PD sample was 11.42 years (*SD* = 4.4), and 12 years of education was used as a cut-off to compare more educated and less educated patients. The side of onset of symptoms information was missing for five patients.

The healthy controls were recruited from among the patient's relatives or staff at the hospital. None had a neurological, psychiatric or physical illness, or history of head injury, alcohol or drug abuse. Fifty (22 male; 48 right-handed) controls participated in the study with a mean age of 57.68 years (*SD* = 3.2) and mean education of 11.96 (*SD* = 3.2). The mean MMSE was 28.66 (*SD* = 0.9). Informed consent was obtained from the patients and controls.

### 2.2. Assessments and procedures

None of the patients was demented and none had scores below the cutoff of 24 on the Mini-Mental Status Examination (MMSE) (mean MMSE = 28.03, *SD* = 1.9) [9]. The Hamilton Depression Scale [HAM-D; 10] was used to assess depression. A semi-structured interview is used to detect the presence of depression with this test involving 17 items (18 to 21 are optional), each with 3 to 5 possible response categories. Scores range from 0 to 60, with higher scores indicative of more severe depression. Total scores of 18 or less indicated absence of depression, 19 to 22 mild depression, 23 to 25 moderate depression and higher than 26 severe depression. The mean HAM-D score of the patients was 15.31 (*SD* = 10.6). The Frontal Assessment battery (FAB) [11] provided a short and easily administered measure of executive dysfunction and 'frontal' behaviour disorders. The six items of the FAB assess conceptualization (similarities), mental flexibility (phonemic verbal fluency), motor programming, sensitivity to interference, inhibitory control, and environmental autonomy. FAB scores range from 0 to 18, with lower scores indicative of greater 'frontal' executive deficits. The patients were assessed on their usual dose of medication, and were in the 'on' state during the assessment.

All participants completed a phonemic and a semantic verbal fluency task [12]. For the semantic fluency task the participant had to generate as many different types of animals as possible during 60 seconds. In the phonemic fluency task, participants were required to generate as many words as possible starting with the letter "F" in 60 seconds excluding proper nouns. The examiner wrote down the words generated and the score was the total number of correct words generated after exclusion of repetitions and errors. The assessments were completed by the same qualified person (EC) in a quiet and well-lit room.

The study was approved by the Cuban National Ethical Committee. Informed consent was obtained from all participants.

Statistical analysis with SPSS version 16.0 was used to check for normal distribution of data (using Shapiro-Wilk tests and looking at the symmetrical distribution of data points) and to carry out the comparisons between groups, using one-way analysis of variance (ANOVA), independent samples $t$-tests for post-hoc comparisons, Pearson and Spearman (Hoehn & Yahr) correlation coefficients and multivariate regression analysis.

## 3. Results

The patient and control groups did not differ significantly in terms of age [$t_{(348)} = 0.17, p = 0.86$], or education [$t_{(348)} = -0.81, p = 0.41$]. The sex distribution differed in the two groups [$\chi_{(1)} = 46.81$, $p > 0.001$] with a greater number of male than female participants in the PD than the control group. While none of the patients was demented, nevertheless their score in the MMSE was significantly lower than that of the controls [$t_{(113.4)} = 2.35$, $p = 0.02$]. On the FAB, the mean score for the patients was 12.95 (*SD* = 3.9), and 29.3% of the sample had scores below 12.

Table 1
The results of the analyses of variance (ANOVAs) examining the effect of the clinical and socio-demographic variables on semantic verbal fluency

| | Main effect | Results |
|---|---|---|
| PD vs. C | $F_{(1,349)} = 48.64, p < 0.001$ | *Controls: M: 21.46, SD: 4.2*<br>*PD: M: 15.72, SD: 5.5*<br>*p < 0.001* |
| H&Y (1-4) | $F_{(1,299)} = 16.23, p < 0.001$ | *A priori contrasts show significant linear trend* |
| HAM-D | $F_{(1,299)} = 11.20, p = 0.001$ | *Depressed: M: 14.77, SD: 5.2*<br>*Non-depressed: M: 16.86, SD: 5.3, p = 0.001* |
| Side of onset of symptoms | $F_{(1,296)} = 14.11, p < 0.001$ | *Right onset: M: 14.59, SD: 4.9*<br>*Left onset: M: 16.92, SD: 5.6*<br>*p < 0.001* |
| Age of onset | $F_{(1,299)} = 1.21, p = 0.16$ | Ns |
| Duration of illness | $F_{(1,299)} = 1.40, p = 0.10$ | Ns |
| Gender | $F_{(1,349)} = 0.01, p = 0.92$ | Ns |
| Age | $F_{(1,349)} = 14.40, p = 0.03$ | *Less 58: M: 17.67, SD: 5.3*<br>*Over 58: M: 15.38, SD: 5.9*<br>*p < 0.001* |
| Education | $F_{(1,349)} = 30.62, p < .001$ | *Less 12: M: 15.03, SD: 5.2*<br>*Over 12: M: 18.31, SD: 5.8*<br>*p < 0.001* |

PD vs. C and gender analysis was performed with both groups.
Age analysis was based on median split of age of 58 years, with 152 and 148 patients respectively under and over 58 years old in the patient group For controls, 25 were youngerthan 58 and 25 were above 58 years old.
165 patients had less than 12 years of education and 135 had over 12 years of education . For controls, 24 had less than 12 years of education and 26 had over 12 years of education.
125 patients were non-depressed (HAMD scores of 18 or less) and 175 showed depression (HAMD scores of 19 or above).
159 patients had right onset of symptoms and 136 left onset.
Ns = not significant.
PD = Parkinson's disease.
LC = contrals.

The results of ANOVAs are summarized in Tables 1 and 2. The main measure of interest was the number of words generated in the semantic and the phonemic fluency tasks. A greater number of words was generated in the semantic than the phonemic fluency tasks across both groups (Fig. 1) and the control group generated significantly more words than the patients (Fig. 1 and Table 1).

A series of one-way ANOVAs were completed to examine the effects of PD, H&Y stage of illness, depression, duration of illness, and side of onset of symptoms as well as gender, age and education on verbal fluency measures. PD patients had significantly lower semantic and phonemic verbal fluency scores than the age and education-matched healthy controls. Of the clinical variables, stage of illness (more advanced stage of illness, worse verbal fluency, Fig. 2), and side of onset (right hemibody onset, worse verbal fluency, Fig. 3) and depression (worse semantic verbal fluency when depression present, Table 1) but not age of onset affected verbal fluency scores (Tables 1, 2). Education and age were the only socio-demograpic measures that influenced both verbal fluency scores. Hence, the more educated and the younger had better verbal fluency scores.

### 3.1. Correlations and regression analyses

In the PD group we studied the relationship between semantic and phonemic fluency with clinical and socio-demographic variables. Fluency scores showed significant positive correlations with education [semantic, $r = 0.33$; $p < 0.001$; phonemic, $r = 0.33$; $p < 0.001$], MMSE [semantic, $r = 0.58$; $p < 0.001$; phonemic, $r = 0.56$; $p < 0.001$], and all the subscales from the FAB [semantic, $r = 0.57$, $p < 0.001$; phonemic, $r = 0.60$, $p < 0.001$]. Significant but negative correlations with age [semantic, $r = -0.25$; $p < 0.001$; phonemic, $r = -0.19$; $p = 0.001$], age of onset [semantic, $r = -0.18$; $p = 0.001$; phonemic, $r = -0.15$; $p < 0.01$], H&Y [semantic, $r = -0.41$; $p < 0.001$; phonemic, $r = -0.33$; $p < 0.001$], and the HAM-D scale [semantic,

Table 2
The results of the analyses of variance (ANOVAs) examining the effect of the clinical and socio-demographic variables on phonemic verbal fluency

| | Main effect | Results |
|---|---|---|
| PD vs. C | $F_{(1,349)} = 50.61, p < 0.001$ | *Controls: M: 15.58, SD: 3.3*<br>*PD: M: 10.23, SD: 5.1*<br>*p < 0.001* |
| H&Y (1-4) | $F_{(1,299)} = 11.64, p < 0.001$ | *A priori contrasts show significant linear trend* |
| HAM-D | $F_{(1,299)} = 3.11, p = 0.08$ | |
| Side of onset of symptoms | $F_{(1,299)} = 22.78, p < 0.001$ | *Right onset: M: 8.93, SD: 4.5*<br>*Left onset: M: 11.63, SD: 5.1*<br>*p < 0.001* |
| Age of onset | $F_{(1,299)} = 0.80, p = 0.82$ | Ns |
| Duration of illness | $F_{(1,299)} = 1.37, p = 0.12$ | Ns |
| Gender | $F_{(1,349)} = 0.13, p = 0.71$ | Ns |
| Age | $F_{(1,349)} = 11.81, p < 0.001$ | *Less 58: M: 11.94, SD: 5.3*<br>*Over 58: M: 10.03, SD: 5.0*<br>*p < 0.001* |
| Education | $F_{(1,349)} = 34.60, p < 0.001$ | *Less 12: M: 9.54, SD: 4.8*<br>*Over 12: M: 12.71, SD: 5.1*<br>*p < 0.001* |

PD vs. C – Group and gender analysis was performed with both groups.
Age analysis was based on median split of age of 58 years, with 152 and 148 patients respectively under and over 58 years old in the patient group For controls, 25 wereyounger than 58 and 25 were above 58 years old.
165 patients had less than 12 years of education and 135 had over 12 years of education . For controls, 24 had less than 12 years of education and 26 had over 12 years of education
125 patients were non-depressed (HAMD scores of 18 or less) and 175 showed depression (HAM-D scores of 19 or above).
Ns = not significant.
PD = Parkinson's disease.
LC = contrals.

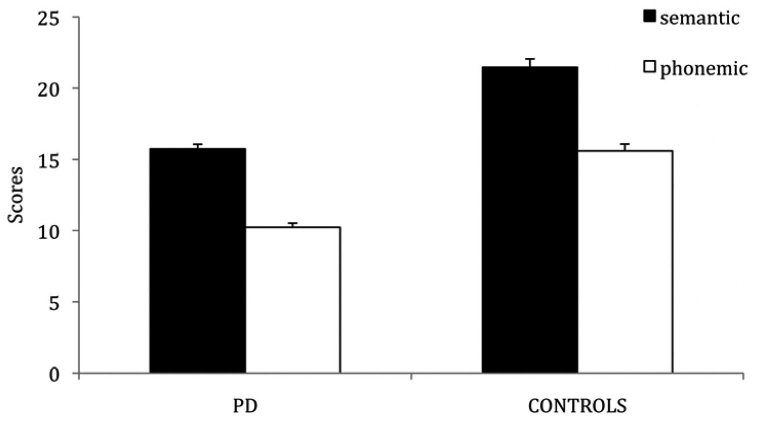

Fig. 1. Mean semantic and phonemic verbal fluency scores for PD and control groups. The bars show the total scores, higher scores signify better performance. Error bars depict standard errors.

$r = -0.19; p = 0.001$] were observed. The correlation between HAM-D and phonemic fluency approached significance [$r = -0.11; p = 0.057$]. None of the correlations between semantic or phonemic fluency and the other measures of interest were significant.

To identify the variables that contributed to the variance of phonemic and semantic fluency in the PD group, two multiple regression analyses were performed. In the first regression analysis, the dependent variable was semantic fluency and the independent variables entered as potential predictors were age, education, MMSE, FAB (total score without the VF item) HAM-D, H&Y,

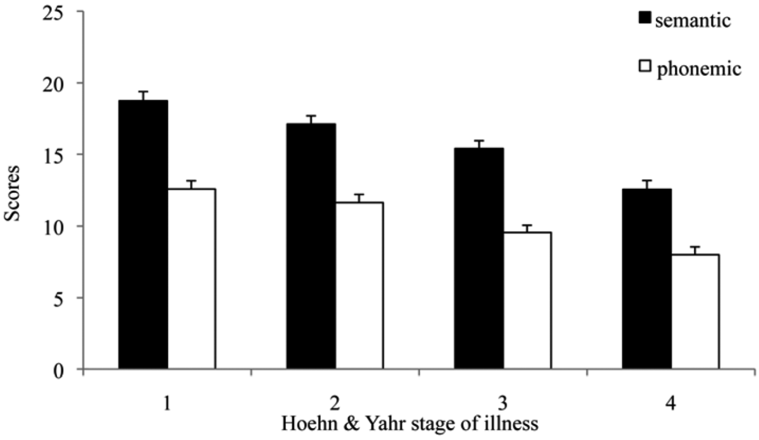

Fig. 2. The effect of Hoehn & Yahr stage of illness on mean semantic and phonemic scores for the PD group. The bars show the total score, higher scores signify better performance. Error bars depict standard errors.

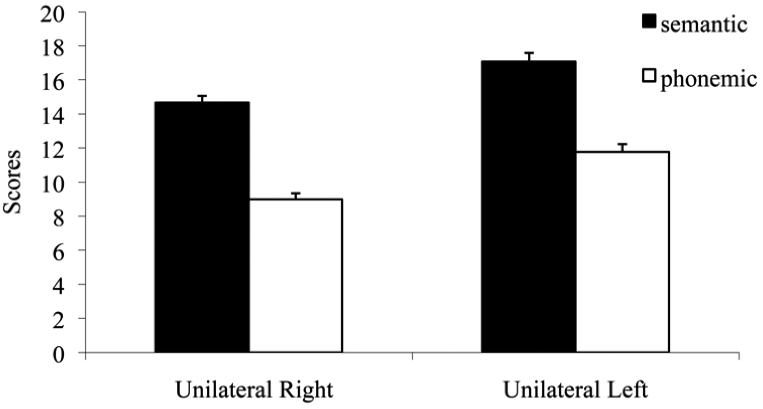

Fig. 3. The effect of side of onset of motor symptoms on mean semantic and phonemic scores. The bars show the total score, higher scores signify better performance. Error bars depict standard errors.

side of onset of symptoms, duration of illness, and age of onset. The MMSE [beta = 0.35, $t_{(299)} = 4.86, p < 0.001$], FAB [beta = 0.19, $t_{(299)} = 2.89, p < 0.\ 01$] and side of onset [beta = 0.14, $t_{(299)} = 2.99, p < 0.01$] predicted 37% (adjusted $R^2$) of the variance of semantic fluency in PD [$F_{(5,294)} = 22.81, p < 0.001, R^2 = 0.37$].

For phonemic fluency, MMSE [beta = 0.38, $t_{(299)} = 5.25$, $p < 0.001$], FAB [beta = 0.19, $t_{(299)} = 2.89$, $p < 0.01$] and side of onset [beta = 0.19, $t_{(299)} = 4.01$, $p < 0.001$] emerged as significant predictors [$F_{(5,294)} = 22.24$, $p < 0.001$, $R^2 = 0.36$] and accounted for 36% (adjusted $R^2$) of the variance.

## 4. Discussion

The main aim of the study was to examine the effect of clinical and demographic variables on verbal fluency in PD. In this respect, we found that stage of illness had a linear effect on verbal fluency that deteriorated with illness progression. In addition, presence of depression was associated with worse verbal fluency scores. Those with onset of PD on the right hemibody had significantly worse verbal fluency than those with left-sided onset. While older age and lower educational levels were associated with significantly poorer semantic and phonemic verbal fluency, gender did not affect verbal fluency scores. Regression analyses revealed global cognitive functioning and executive functioning as respectively assessed by the MMSE and the FAB as significant predictors of both semantic and phonemic verbal fluency in PD. In addition, side of onset also emerged as a predictor of both semantic and phonemic fluency performance in PD.

As expected and consistent with previous studies, PD patients produced significantly fewer words than controls on both versions of the verbal fluency task [2, 13,14]. Larger deficits in phonemic compared to semantic fluency were found in PD patients in some previous studies [15,16] supporting the notion that PD patients show impairment because executive functions are implicated in the phonemic task. In contrast, others have found greater impairment of semantic fluency compared to phonemic fluency in PD [17,18]. Auriacombe et al. [17] proposed that the semantic verbal fluency deficit in PD may be linked to specific problems with retrieval of semantic information. In an attempt to resolve previous inconsistencies, in a meta-analysis with 2644 PD patients from 68 studies Henry and Crawford [19] reported that PD patients showed greater deficits on semantic than phonemic fluency, but that the differences between the two tasks was small. Henry & Crawford [19] suggested that while both phonemic and semantic verbal fluency deficits in PD may relate to the need for effortful retrieval required in both tasks, due to differences in depth of encoding, additional retrieval mechanisms may be required for semantic fluency which may explain the slightly greater deficits on semantic fluency in PD. In the present sample, with the specific verbal fluency tasks that we used, PD patients generated fewer words on phonemic fluency than semantic fluency relative to healthy controls. However, in our view direct comparison of the magnitude of deficits in phonemic and semantic fluency in PD across studies is problematic and misleading. This is because the precise nature of the verbal fluency deficits depends on the particular versions of the verbal fluency tasks used, as previously demonstrated by Azuma et al. [16]. Furthermore, presence/absence and severity of depression across PD samples may also affect the relative pattern of phonemic vs semantic verbal fluency deficits, as demonstrated by our results that depression affects performance on the two fluency tasks.

As an alternative to executive dysfunction or problems with effortful retrieval of semantic information, verbal fluency deficits in PD could also be associated with difficulties in response selection through inhibition of retrieval of inappropriate words in this disorder. According to the model of verbal fluency suggested by Frith et al. [20] verbal fluency is achieved through the modulatory influence of the left dorsolateral prefrontal cortex on the left superior temporal cortex to inhibit generation of inappropriate words (e.g. words highly associated by meaning rather than words starting with the letter 'F' during phonemic fluency) and to allow selection of words that meet the instructions. Deficits in response selection through inhibition of competing responses have been documented in PD [21].

The significant decline of verbal fluency with stage of illness has been previously reported [15,22] and has important implications for the studies which have found deterioration of verbal fluency following STN DBS surgery (for review see [5]). This post-surgical decline in verbal fluency in PD may be due to surgical microlesions affecting cortical-basal ganglionic circuits involved in word retrieval processes [23–25]. Others have shown that the decline in verbal fluency may be a result of an inability to switch from one semantic or phonemic subcategory to another during verbal fluency [26]. In light of this effect of stage of illness on verbal fluency, inclusion of an unoperated control patient group is essential for unequivocal interpretation of the deterioration of verbal fluency after surgery, as has now been established in a number of controlled studies [27,28]. Our results have shown that depression (HAM-D), global cognitive ability (MMSE) and executive dysfunction (FAB) significantly influence verbal fluency performance in PD. Therefore, since the presence/absence and/or severity of depression or executive dysfunction can potentially change with DBS of the STN [29], it is important to investigate the influence of such changes in depression and executive function with surgery on the documented decline of verbal fluency following surgery [5] in future studies.

The present sample had a large depressed subgroup that showed worse semantic and phonemic fluency than those without depression, indicating that in PD depression negatively influences tasks requiring word generation. Troster et al. [30] also compared cognition in PD patients with and without depression and reported lower verbal fluency in the depressed group compared to the non-depressed. Other studies [31] obtained different results from ours and those of Troster et al. [30], and found that depression did not affect verbal fluency performance, perhaps either due to the smaller sample size or less severe depressive state, or the specific nature of the verbal fluency tasks used.

The side of onset of the motor symptoms affected scores on both verbal fluency measures and emerged as a predictor of verbal fluency performance. Patients with a right onset of the disease generated significantly fewer words compared to those with left onset of the symptoms (see Fig. 3). This may reflect the left hemispheric specialization of language. In fact, as noted above, imaging studies have shown activation of the left dorsolateral prefrontal cortex and the left temporal cortex during phonemic verbal fluency relative to word generation [20]. Similarly, Tower et al. [32] assessed a group of 88 PD patients with a unilateral onset of symptoms and found worse performance on cognitive tasks including the verbal fluency test, in patients with right hemibody onset of motor symptoms thus supporting our results.

As expected, the younger and the more educated patients did better compared to the older or less educated patients on the verbal fluency tests. This was also confirmed by the negative association between age and verbal fluency scores and a positive association between years of education and verbal fluency. Findings from other studies evaluating semantic and phonemic verbal fluency have revealed better performance on both tasks with greater educational level and for younger participants [31]. Similar to Lanting et al. [33] we did not find any significant gender effects on verbal fluency.

Some previous studies have shown that in PD performance of verbal fluency is improved when patients are assessed on than off levodopa medication [2]. Our sample was assessed on medication. It would be of interest to assess the impact of levodopa medication and any possible interaction with other clinical or demographic variables on verbal fluency performance in future studies.

In conclusion, our main finding is that both the motor features of PD such as side of onset of the motor symptoms and stage of illness as well as the non-motor features such as depression (HAM-D) and global cognitive ability (MMSE) and executive dysfunction (FAB) influence verbal fluency performance of patients. Our results with this large sample also confirm that demographic variables such as age and educational level which influence verbal fluency in healthy controls, also have an impact on the verbal fluency performance of PD patients.

## Acknowledgments

We are grateful to all the participants. This research was supported by a PhD studentship from Fundación Caja Madrid, Spain (IO), and a Royal Society Travelling Fellowship.
